# Iron deficiency in patients with cardiogenic shock: protocol for a scoping review

**DOI:** 10.1136/bmjopen-2024-092891

**Published:** 2025-04-19

**Authors:** Lorenzo Germinario, Daniel Catena, Sascha Ott, Tobias Roeschl, Yassine Ghamri, Alexander Meyer, Benjamin O’Brien, Felix Schoenrath

**Affiliations:** 1 Department of Cardiac Anesthesiology and Intensive Care Medicine, Deutsches Herzzentrum der Charité - Medical Heart Center of Charité and German Heart Institute Berlin, Berlin, Germany; 2 Charité – Universitätsmedizin Berlin, corporate member of Freie Universität Berlin and Humboldt-Universität zu Berlin, Campus Virchow-Klinikum, Augustenburger Platz 1, 13353, Berlin, Germany; 3 DZHK (German Centre for Cardiovascular Research), Partner Site Berlin, 13353, Berlin, Germany; 4 Department of Anesthesiology, Outcomes Research Consortium, Cleveland Clinic, Cleveland, OH 44195, Ohio, USA; 5 Department of Cardiothoracic and Vascular Surgery, Deutsches Herzzentrum der Charité - Medical Heart Center of Charité and German Heart Institute Berlin, Berlin, Germany; 6 Berlin Institute of Health, Berlin, Germany; Charité-Universitätsmedizin Berlin, Institute of Medical Informatics, Invalidenstraße 90, 10115, Berlin, Germany; 7 Department of Perioperative Medicine, St Bartholomew’s Hospital and Barts Heart Centre, London EC1A 7BE, UK

**Keywords:** Heart failure, Other metabolic, e.g. iron, porphyria, Adult intensive & critical care, Coronary heart disease

## Abstract

**Abstract:**

**Introduction:**

Cardiogenic shock (CS) is a severe condition characterised by low cardiac output and often hypotension, which results in organ hypoperfusion due to cardiac failure. As a form of acute heart failure, this condition seems to share similar underlying pathological mechanisms. It is well established that iron deficiency is correlated with chronic and acute heart failure, causing worsening of the symptoms, reduction of quality of life and survival and simultaneously increasing the rehospitalisation rates for all causes in these patients. It remains unclear whether there is an association between iron deficiency and CS. The objective of this scoping review will be to determine the actual state of the art regarding the significance of iron deficiency in patients affected by CS.

**Methods and analysis:**

We will conduct a systematic review of the literature using MEDLINE and EMBASE via ‘Ovid’ (Elsevier) and Web of Science (2024 Clarivate). The goal is to analyse the incidence and clinical significance of iron deficiency in patients affected by cardiogenic shock. To gain a deeper insight into the underlying pathophysiological mechanisms, the review will include basic research conducted on both human subjects and on animal models as well as observational, randomised controlled studies and systematic reviews and meta-analysis. To maximise the identification of relevant reports and reduce loss of information, a systematic search of the literature will be performed from inception until January 2025 using the terms “iron deficiency” as well as “iron”, “ferritin”, “transferrin”, “transferrin saturation”, “hepcidin” and “soluble transferrin receptor” matching these terms with the keywords “cardiogenic shock”, “acute heart failure”, “advanced heart failure”, “decompensated heart failure”, “lvad”, “left ventricular assist device”, “mechanical circulatory support”, “VA-ECMO” and “Extracorporeal Life Support”. We will also use the corresponding MeSH and Emtree terms. In order to find grey literature, we will use the OADT.org internet-based database.

**Ethics and dissemination:**

No additional ethics approval is required, as this review is based on existing research without new data collection. Only studies with ethics approval will be included. We plan to publish our findings in a peer-reviewed journal and present them at international conferences on cardiology, intensive and acute cardiovascular care, cardiac surgery and cardioanaesthesiology.

**STRENGTHS AND LIMITATIONS OF THIS STUDY**

- Common limitations of a scoping review are the potential loss of information deriving from grey literature, reports in languages other than English or reports that are not included in the used medical report databases.
- This scoping review follows a broad spectrum research strategy, utilising multiple search terms, multiple databases and forward and backward citation searches to identify missing reports.
- We will include articles published in languages other than English and conduct research on a specific database for grey literature.
- We will use established methodologies and tools to search the literature, select the reports, extract the data and assess bias in the single studies, even if not methodologically typical for scoping reviews.
- Both human and animal studies will be included to assess clinical as well as pathophysiological aspects of iron deficiency in CS.

This Scoping Review Protocol was written following the ‘JBI Manual for Evidence Synthesis, Chapter 11: Scoping Reviews’^1^ and is being reported in accordance with the ‘PRISMA Extension for Scoping Reviews (PRISMA-ScR): Checklist and Explanation’^2^

In case of changes in the protocol, we will report this, mentioning the rationale for the changes.

The scoping review will be sustained by the clinic for Cardioanesthesiology and Intensive Medicine as well as by the clinic for Heart, Thoracic and Vascular Surgery of the ‘Deutsches Herzzentrum der Charité–Universitätsmedizin’ Berlin; Germany.

## Introduction

### Rationale

 Cardiogenic shock (CS) is a severe and acute form of heart failure (HF) characterised by hypoperfusion of vital organs due to a reduction of the cardiac output, which is secondary to different pathological mechanisms. Various definitions have been applied to this condition,^3^,^7^ including both clinical and haemodynamical parameters. Recently, these more (invasive) haemodynamic definitions shifted towards clinical definitions in order to allow easier diagnosis and a staging of the severity of CS was introduced by the ‘Society for Cardiovascular Angiography and Interventions’ (SCAI) in 2019 (SCAI-Classification),^8^ which was shown to strongly correlate with the mortality both in the cardiac^9^ and in the cardiac surgery intensive care unit.^10^

CS is defined as a condition in which persistent hypotension (systolic blood pressure less than 90 mm Hg or mean blood pressure less than 65 mm Hg for usually 30 min or longer) or the need for catecholamines is correlated with signs and symptoms of hypoperfusion, including oliguria, altered mental status, cool extremities and pulmonary congestion (in case of left ventricular failure). Despite advances in the management of underlying conditions, including coronary interventions and drug-based therapies, the incidence of CS has increased in the past decades^11^,^13^ and the outcome remains poor with mortality rates ranging from 20% to 40% depending on the phenotype and severity of CS.^10^,^1214 15^

Iron deficiency is a highly prevalent condition among patients affected by chronic and acute HF. Many observational studies have suggested a correlation between iron deficiency and poor outcomes, including reduced quality of life and high rehospitalisation rates.^16^,^19^ However, the precise mechanism through which iron deficiency contributes to cardiac pathology remains unclear. Intense research has been conducted on this topic, and randomised controlled trials have shown the efficacy of iron substitution in patients affected by both chronic^20^,^22^ and acute^23 24^ HF. For this reason, iron implementation was introduced as a therapy with a level of evidence IIb in the ‘2016 ESC guidelines for diagnosis and management of acute and chronic heart failure’^6^ and a level of evidence 2 a in the ‘2022 AHA/ACC/HFSA Guideline for the Management of Heart Failure: A Report of the American College of Cardiology/American Heart Association Joint Committee on Clinical Practice Guidelines’^25^ in patients with chronic HF and after acute decompensation of HF.

### Objectives

The objective of this scoping review will be to examine the current literature to define the association between iron deficiency and the most severe form of HF, i.e. CS, and find similarities to the established relationship between iron deficiency and the other forms of HF.

## Methods and analysis

### Searches

We will perform a systematic review of literature published from inception until January 2025 using MEDLINE and EMBASE via ‘Ovid’ (Elsevier) and Web of Science (2024 Clarivate) databases. Additionally, we will include posters presented at international congresses that have not been published in journals, as well as grey literature using OATD.org (Open Access Theses and Dissertations), an international internet-based thesis and dissertations archive.

### Search strategies and inclusion criteria

The search will be conducted by two independent reviewers on the same date (LG and DC).

#### Search strategy for MEDLINE and EMBASE

We will primarily perform a phrase search using the terms

- *iron deficiency.ti,ab,kw.*
- *iron.ti,ab,kw.*
- *ferritin.ti,ab,kw.*
- *transferrin.ti,ab,kw.*
- *transferrin saturation.ti,ab,kw.*
- *hepcidin.ti,ab,kw.*
- *soluble transferrin receptor.ti,ab,kw*.

matched with the Boolean logic term ‘OR’. Same search strategy will be done with the terms

- *cardiogenic shock.ti,ab,kw*.
- *acute heart failure.ti,ab,kw*.
- *advanced heart failure.ti,ab,kw*.
- *decompensated heart failure.ti,ab,kw*.
- *lvad.ti,ab,kw*.
- *left ventricular assist device.ti,ab,kw*.
- *mechanical circulatory support.ti,ab,kw*.
- *VA-ECMO.ti,ab,kw*.
- *Extracorporeal Life Support.ti,ab,kw*.

The results of the two searches will be matched with the Boolean logic operator ‘AND’ for outsource reports which do not present the combination of both searches.

We will also search the literature using the following ‘Mesh-Terms’ and ‘Emtrees’ and connect them through Boolean logic operator OR:

- *Iron Deficiencies/*
- *Transferrin/*
- *Receptors, Transferrin/*
- *Ferritins/*
- *Hepcidins/*
- *Iron/*

The totality of results will be matched with Boolean logic operator AND with the following terms:

- *Shock, Cardiogenic/*
- *Heart Failure/*
- *Myocardial Infarction/*
- *Cardiomyopathy, Dilated/*
- *Heart Transplantation/*
- *Ventricular Dysfunction, Left/*
- *Heart-Assist Device/*
- *Extracorporeal Membrane Oxygenation/*
- *Assisted Circulation/*

#### Search strategy for Web of Science

1. ((TS=cardiogenic shock)) OR TS=(acute heart failure) OR TS=(advanced heart failure) OR TS=(decompensated heart failure) OR TS=(LVAD) OR TS=(left ventricular assist device) OR TS=(VA ECMO) OR TS=(mechanical circulatory support) OR TS=(extracorporeal life support)
2. ((TS=iron deficiency)) OR TS=(iron) OR TS=(transferrin) OR TS=(transferrin saturation) OR TS=(soluble transferrin receptor) OR TS=(ferritin) OR TS=(hepcidin)
3. #1 AND #2

#### Search strategy for OATD.org

abstract: (“cardiogenic shock”, OR “acute heart failure”, OR “decompensated heart failure”, OR “advanced heart failure”, OR “lvad”, OR “left ventricular assist device”, OR “mechanical circulatory support”, OR “VA ECMO”, OR “extracorporeal life support”) AND abstract: (“iron deficiency”, OR “ferritin”, OR “transferrin”, OR “transferrin saturation”, OR “soluble transferrin receptor”, OR “hepcidin”) and

title: (“cardiogenic shock”, OR “acute heart failure”, OR “decompensated heart failure”, OR “advanced heart failure”, OR “lvad”, OR “left ventricular assist device”, OR “mechanical circulatory support”, OR “VA ECMO”, OR “extracorporeal life support”) AND title: (“iron deficiency”, OR “ferritin”, OR “transferrin”, OR “transferrin saturation”, OR “soluble transferrin receptor”, OR “hepcidin”)

title: (“cardiogenic shock”, OR “acute heart failure”, OR “decompensated heart failure”, OR “advanced heart failure”, OR “lvad”, OR “left ventricular assist device”, OR “mechanical circulatory support”, OR “VA ECMO”, OR “extracorporeal life support”) AND title: (“iron deficiency”, OR “ferritin”, OR “transferrin”, OR “transferrin saturation”, OR “soluble transferrin receptor”, OR “hepcidin”)

Once we have identified the reports of interest, we will perform a forward and backward report search using Citationchaser.^26^

We will select basic research articles on both human and animal models to evaluate the pathophysiological basis of iron deficiency in CS. Additionally, we will review observational studies (retrospective and prospective, cohort, case-control, cross-selection studies, etc.), randomised controlled trials on iron substitution in patients affected by CS and acute or acutely decompensated HF and systematic reviews and meta-analysis if the provided information is relevant to the aim of the scoping review.

The definition used for diagnosing iron deficiency is very inhomogeneous through the literature. We will not prioritise a specific definition over another and will include all reports independently of the definition chosen by the authors.

Iron deficiency is often associated with anaemia, a comorbidity known to impact prognosis and outcomes of HF patients. In order to distinguish between the impact of iron deficiency and anaemia, we will include all reports explicitly stating that the researched comorbidity is iron deficiency. Reports on iron deficiency-dependent anaemia will be included with a specific mention. Reports analysing the effect of anaemia without mentioning iron deficiency or those considering forms of anaemia different than iron deficiency-associated anaemia will be excluded.

Articles written in any language will be considered. In case of reports not written in English, German or Italian, we will consider eligibility only if a coherent translation can be provided by automatic language translation tools (eg, Google Translate).

Comments, editorials, narrative reviews, case reports, as well as economic analyses will be excluded from the review as they do not align with the objectives of the scoping review.

**Figure 1:**
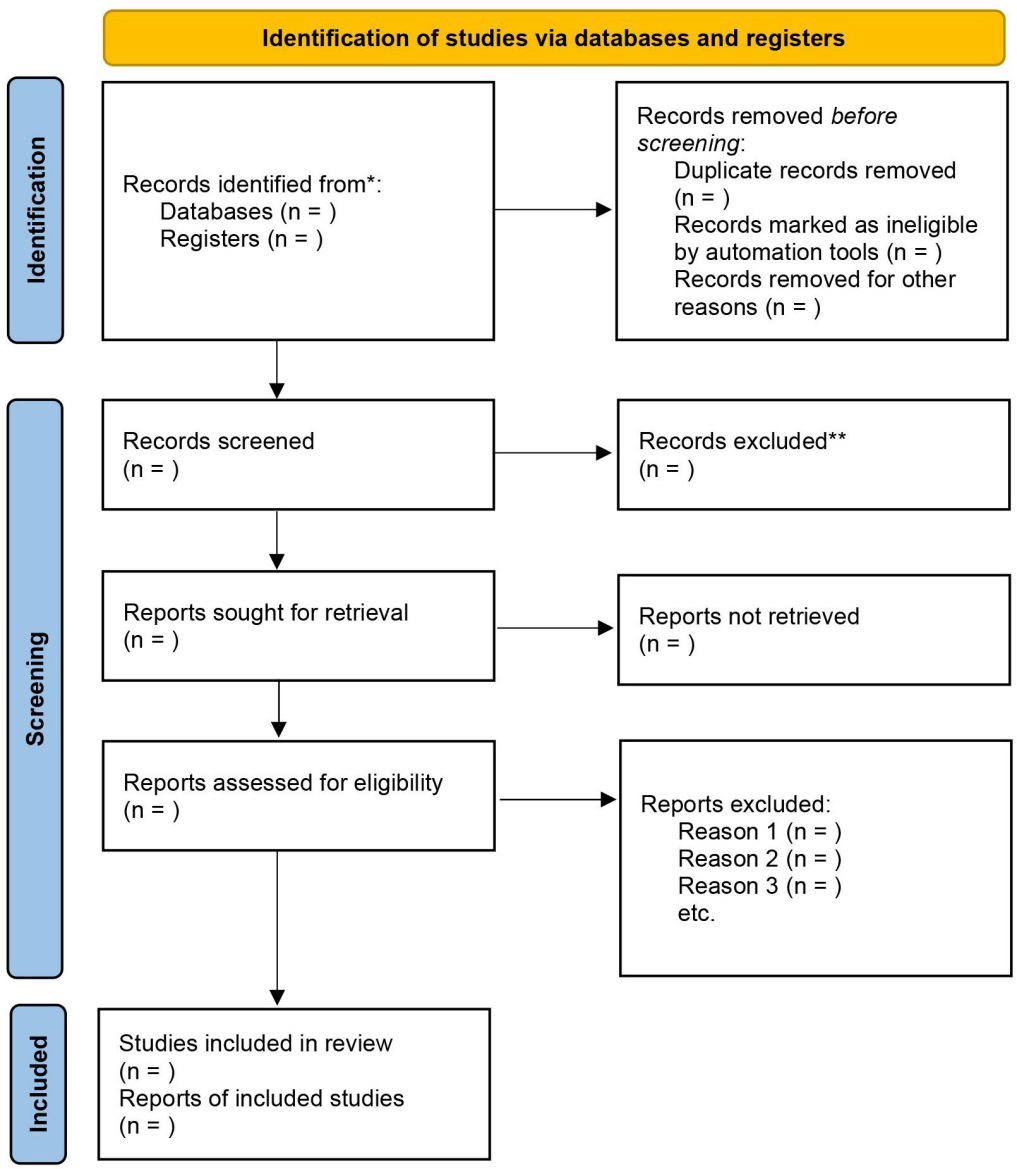
PRISMA 2020 flow diagram for new systematic reviews which included searches of databases and registers only. *Consider, if feasible to do so, reporting the number of records identified from each database or register searched (rather than the total number across all databases/registers). **If automation tools were used, indicate how many records were excluded by a human and how many were excluded by automation tools. Source: Page *et al*.^27^

In order to provide a figurative explanation of the included and excluded reports, we will use the PRISMA 2020 flow diagram for new systematic reviews^27^ as shown in figure 1.

### Outcome measures

In observational and randomised controlled studies and in systematic reviews and meta-analysis, the primary endpoint will be the incidence of iron deficiency in populations with CS. Secondary endpoints will include difference in 30-day, in-hospital and ICU mortality, SCAI stage, duration of ICU and hospital stay, use of mechanical circulatory support, duration of mechanical circulation as well as use and duration of mechanical ventilation in patients with versus without iron deficiency.

For articles on basic research on animals and human models, endpoints will focus on the pathophysiological association between iron deficiency and CS or acute, advanced or decompensated forms of HF.

### Duplicates exclusion and report selection

To avoid duplications in the researched data, we will use web-based duplicate identification tools such as ‘Systematic Review Accelerator’^28 29^ (Bond University and Institute for Evidence-Based Healthcare, Robina, Australia), Sciwheel (2022 Technology for Sage) and EndNote (2024 Clarivate). The selected reports will be screened by title and abstract, and non-pertinent reports will be excluded. Once all reports assessed for eligibility are selected, we will perform a selection by full text in order to identify the relevant records and outsource all materials that are inconsistent with the goal of this review. All these steps will be conducted by two independent reviewers (LG and DC).

After identifying all selected studies, we will confront the selected reports using the ‘Disputatron’ tool in the ‘Systematic Review Accelerator’. In case of non-univocal selection, the two screeners will discuss each report in order to decide for selection or exclusion. Further discrepancies will be resolved by a third independent member of the review team.

### Data extraction and analysis

In order to extract the data from the selected articles, we will use a standard Data Collection Form proposed by ‘JBI Manual for Evidence Synthesis, Chapter 11: Scoping Reviews: 11.2.7 Data extraction’,^1^ the ‘Recommendations for the extraction, analysis, and presentation of results in scoping reviews’^30^ as well as the ‘Preferred Reporting Items for Systematic reviews and Meta-Analyses extension for Scoping Reviews (PRISMA-ScR) Checklist’.^2^

The information for data collection will include:

- Author(s).
- Year of publication.
- Origin/Country of origin.
- Aims/purpose.
- Population and sample size (if applicable).
- Methodology/methods.
- Intervention type/duration.
- Outcome measures.
- Key findings that relate to the scoping review question(s).

We will group reports depending on the different definitions used for the diagnosis of iron deficiency, in order to evaluate whether diverse definitions have different impacts on the development and prognosis of CS.

We will primarily analyse reports about the comorbidity ‘iron deficiency’ as well as articles about iron deficiency-associated anaemia or iron deficiency plus anaemia. We will report results separately depending on the comorbidity, in order to reduce the risk of bias due to the confounding effect of anaemia on the prognosis of these patients or cross-effects of one comorbidity on the other.

We will use graphic representation in order to divide the reports depending on the definition of iron deficiency and number of comorbidities (iron deficiency alone or anaemia with iron deficiency).

After the identification of all selected studies, we will subdivide them into the following topics: ‘basic research on animal models’, ‘basic research on human models’, ‘observational studies’ (both retro- and prospective) and ‘intervention trials and systematic reviews/meta-analysis’. We will perform a descriptive, narrative analysis of the extracted information from the selected reports. Special attention will be given to the funding source of the studies and potential conflicts of interest of the authors.

In case of missing or unclear study data, we will contact the authors of the original articles for further information.

Contact through electronic mail will be attempted up to a maximum of three times.

### Risk of bias

Generally, the determination of risk of bias is not typical in the methodology of scoping reviews. Nevertheless, we decided to include this additional evaluation (as established by tool 12 of the ‘Preferred Reporting Items for Systematic reviews and Meta-Analyses extension for Scoping Reviews (PRISMA-ScR) Checkliste’^2^) to provide the highest quality of evidence for our scoping review.

In order to assess the possible risk of bias for each report, if applicable in the specific case, we will collect information using the Cochrane Collaboration tool for assessing the risk of bias (Table 8.5.a in the Cochrane Handbook for Systematic Reviews of Interventions), which covers sequence generation, allocation concealment, blinding, incomplete outcome data (eg, dropouts and withdrawals) and selective outcome reporting. We will divide the reviewed articles into high and low risk of bias. In case of insufficient information, we will define the risk of bias as uncertain and contact the authors for further information.

In order to evaluate the risk of bias for observational studies, we will use the ROBINS-I tool^31^ for intervention studies and the ROBINS-E tool^32^ for exposure studies.

For articles reporting animal studies, we will use the ‘SYRCLE’s risk of bias for animal studies. This tool was developed based on the Cochrane RoB tool and was adjusted for specific risk of bias that plays a role in animal intervention studies.^33^

In order to evaluate the quality of systematic reviews, we will use the AMSTAR two tool.^34^

Two reviewers (LG and DC) will carry out this process independently to minimise bias in the analysis.

We plan to analyse all studies we will find in the literature search and will highlight the bias risk (high, low or uncertain) without omitting any result.

### Patient and public involvement

No patient representatives or members of the public were directly involved in the planning, design, conduct or reporting of this protocol, and no primary data were collected.

## Ethics and dissemination

No additional ethics approval process will be required for this research, as this review analyses and synthesises existing research rather than conducting new data collection.

We will only include studies which underwent an ethical approval process in the institution where the research was performed.

We plan to present the results of our scoping review in a peer-reviewed journal and at international conferences regarding cardiology, intensive and acute cardiovascular care, cardiac surgery and cardioanaesthesiology.
