# Supplementary material for: Iron deficiency in patients with cardiogenic shock: protocol for a scoping review
Source: BMJ Open. 2025 Apr 19;15(4):e092891. doi: 10.1136/bmjopen-2024-092891 (PMC12010308; doi:10.1136/bmjopen-2024-092891)
Supplement: Supplementary file 1 [file bmjopen-2024-092891.reviewer_comments.pdf]

PEER REVIEW HISTORY

BMJ Open publishes all reviews undertaken for accepted manuscripts. Reviewers are asked to complete a checklist review form and are provided with free text boxes to elaborate on their assessment. These free text comments are reproduced below.

ARTICLE DETAILS

Title (Provisional)

Iron deficiency in patients with cardiogenic shock: Protocol for a scoping review.

Authors

Germinario, Lorenzo; Catena, Daniel; Ott, Sascha; Roeschl, Tobias; Ghamri, Yassine; Meyer, Alexander; O’Brien, Benjamin; Schoenrath, Felix

---

VERSION 1 - REVIEW

**Reviewer**	**1**
**Name**	**Ray , Robin**
**Affiliation**	**St Georges Hospital, London, UK., Cardiology**
**Date**	**02-Jan-2025**
**COI**	**None**

Thank you for the invitation to review the manuscript for BMJ Open titled "Iron deficiency in patients with cardiogenic shock: Protocol for a scoping review".

The topic of iron deficiency in cardiogenic shock is an important area of research, as it builds upon the established relationship between iron deficiency and heart failure. This scoping review could provide valuable insights into the pathophysiology and management of cardiogenic shock. Iron deficiency is a common comorbidity in heart failure patients, associated with poor outcomes. However, its role in cardiogenic shock remains unclear. The protocol includes a comprehensive search strategy with inclusion of diverse study types. The inclusion of basic research and animal models will help to clarify potential underlying mechanisms linking iron deficiency and cardiogenic shock.

While the protocol is generally well-designed, there are several areas where it could be strengthened.

How will the definition of iron deficiency be addressed in this cohort? The protocol does not specify which definition of iron deficiency will be used and how differing inclusion criteria from different studies will be analysed. Recent evidence suggests that transferrin saturation (TSAT)

<20% may be a more reliable indicator of iron deficiency in heart failure patients than ferritin levels alone. A recent meta-analysis found that patients with TSAT <20% obtained more benefits from intravenous iron with respect to the endpoint of recurrent heart failure hospitalisation and cardiovascular death. However, several studies suggest that high ferritin levels may be associated with worse outcomes in cardiogenic shock. Ferritin levels in cardiogenic shock can be elevated due to inflammation, but may also be low in cases of iron deficiency.

The protocol would benefit from clearly outlining how it will differentiate between iron deficiency with and without anaemia, as these conditions may have distinct implications in cardiogenic shock. Studies have highlighted that anaemia in the absence of iron deficiency might exert a more pronounced influence on outcomes in cardiogenic shock compared to iron deficiency on its own. It is important to address how these components will be separated in the analysis and to explore whether their effects are cumulative. This distinction is important because it may reveal nuanced relationships between iron status, anaemia, and patient outcomes in cardiogenic shock, potentially guiding more targeted therapeutic approaches. An EJHF study by Obradovic et al. (2024) found that anaemia without iron deficiency had a strong impact on 30-day mortality in cardiogenic shock patients, while iron deficiency alone did not significantly affect outcomes. This highlights the complexity of iron metabolism in acute cardiovascular conditions and underscores the need for a comprehensive review of the literature.

The protocol would benefit from specifying primary and secondary outcomes of interest, such as mortality, length of hospital stay, or need for mechanical circulatory support. More detail on how the authors plan to synthesise the data would be helpful, particularly given the diverse study types to be included.

It may also be helpful to include an outline for assessing the quality of included studies to strengthen the protocol e.g. Newcastle-Ottawa Scale for observational studies, SYRCLE's risk of bias tool for preclinical animal studies etc.

This is a well-conceived protocol addressing an important clinical question. With suggestions as above, this scoping review has the potential to make a valuable contribution to the field.

VERSION 1 - AUTHOR RESPONSE

The comments made are of significant importance, and we were very glad to integrate them to improve the protocol.

1) In response to the comment regarding the definition of iron deficiency:
We will not use a pre-set definition and will not prioritize a specific definition over another. We will include all reports independently of the definition chosen by the authors, in order to reduce the information loss derived from different definitions in literature. To provide a clearer insight into the problem of these different definitions, we will group the reports

dependently on the definition used in order to analyse the results according to the definition itself. This approach will enable us to determine if some definition has a predominant influence on the incidence or outcome in patients diagnosed with cardiogenic shock.

2) Regarding the comment on the role of anaemia:
The discrimination between the specific role of iron deficiency compared to the role of anaemia (for example iron deficiency-associated anaemia but also iron independent forms) is very difficult, because the two comorbidities often coexist. We will try to subdivide the selected reports in those in which only iron deficiency is analysed and those in which there is a combination of comorbidities (i.e. ID plus anaemia or ID-associated anaemia). Articles on iron deficiency-dependent anaemia, will be included with a specific mention. Reports analysing the effect of anaemia without mentioning iron deficiency or those considering forms of anaemia different than iron deficiency-associated anaemia will be excluded.

We will provide a visual resume of the reports grouping them on definition and on comorbidities.

3) Regarding the comment on Outcomes:
We have integrated different outcomes in our protocol, as recommended. Primary outcome will be incidence of iron deficiency in patients diagnosed with cardiogenic shock, depending on the definition of the authors. Secondary endpoints will include difference in 30-day-, in-hospital- and ICU-mortality, SCAI stage, duration of ICU- and hospital-stay, use of mechanical circulatory support, duration of mechanical circulation as well as use and duration of mechanical ventilation in patients with vs. without iron deficiency. Basic research reports will be analysed on the effect of iron deficiency on cardiovascular pathophysiology.

4) In response to the comment regarding extending the methodological tools for assessment the risk of bias:
Typically, the methodology of scoping reviews does not foresee the need of assessment of risk of bias, even though the “Preferred Reporting Items for Systematic reviews and Meta-Analyses extension for Scoping Reviews (PRISMA-ScR) Checkliste” provides the possible use of a tool (12) to do so. Risk of bias is a concept that is generally applied to systematic reviews in which data extraction is highly dependent on the quality of the primary data themselves. Nevertheless we decided to add a quality check and an evaluation of critical appraisal in order to provide a scoping review with highest standards and a methodology as near as possible to a systematic review.

We decided to maintain the section “risk of bias” and added, as you recommended, tools to evaluate observational and animal studies. For observational studies we decided to use the ROBINS-I and ROBINS-E tools (for intervention and exposure respectively), because they do not use a numerical scoring system as the Newcastle-Ottawa scale and we think, the analysis of the risk of bias will be more accurate. For animal studies we will use, as advised, the “SYRCLE’s risk of bias for animals studies”. We also added to our materials systematic reviews and meta-analysis. For this reason we will also include the AMSTAR 2 tool for the evaluation of quality of systematic reviews.

As you can see, we did some changes in the searched databases, search strategies, methodology and tools in the revised protocol. This is due to the fact, that we did an intense research in order to optimize the protocol from a methodological and practical perspective.

1) Regarding the statement that the protocol was written following the “Preferred Reporting Items for Systematic reviews and Meta-Analyses extension for Scoping Reviews (PRISMA-ScR) Checkliste”, we decided to change it in:

   This Scoping Review Protocol was written following the “JBI Manual for Evidence Synthesis, Chapter 11: Scoping Reviews“ and is being reported in accordance to the “PRISMA Extension for Scoping Reviews (PRISMA-ScR): Checklist and Explanation”.

   Explanation:
   PRISMA is a reporting guide line and not a manual, so we would like to correct this wrong statement. We here report the protocol in accordance to PRISMA, whilst the protocol was written following the „JBI Manual for evidence synthesis: Chapter 11: Scoping Reviews“.

2) We have decided to change the search interface and one database in our search strategy. We will search MEDLINE using “Ovid” rather than PubMed, because we subjectively prefer the “Ovid” interface and because it seems easier to perform the MEDLINE and the Embase search on one interface. We also decided to exclude ScienceDirect and use Web of Science (© 2024 Clarivate™) as third database, because we are concerned, that using a single publisher’s database would introduce a bias in the selection of the reports. We also added to our search strategy a database for grey literature (OATD.org – Open Access Thesis and Dissertation) in order to scan non peer-reviewed literature. We define how we will perform forward and backward searching, i.e. using the Citationchaser tool. We also have redefined the timeframe for our search starting from the inception of the databases until January 2025.

3) We have provided a much more detailed description of our search strategy including search strings and terms. We will do a double search pro database using phrase search and Mesh/Emtree terms and we decided to use pending subheadings and limit our search fields to title, abstract and authors keywords. We will include systematic reviews and meta-analysis in our search, because the goal of the review is to gain the biggest number and highest quality of information about the topic.

4) We decided to extend the number of tools we will use in the different parts of the review. Web-based tools to help to organize the work from the deduplication of results, to selection of reports and comparison of results after selection will be introduced in the methodology of the protocol. We will for example use the “Systematic Review Accelerator”, a web-based software which is provided by Bond University and Institute for Evidence-Based Healthcare, Robina, Australia or “Citationchaser” a web-based tool to find forward and backward citations.
